# Supplementary material for: The impact of perinatal maternal stress on the maternal and infant gut and human milk microbiomes: A scoping review
Source: PLoS One. 2025 Feb 28;20(2):e0318237. doi: 10.1371/journal.pone.0318237 (PMC11870360; doi:10.1371/journal.pone.0318237)
Supplement: S1 Table 1 — (DOCX) [file pone.0318237.s004.docx]

Table 1: Search terms

|  | Key words for Literature search strategy | | | | |
| --- | --- | --- | --- | --- | --- |
| Searches to match Concepts | | **Population AND** | **Concept 1 AND** | **Concept 2 AND** | **Context** |
| 1. Maternal stress and gut microbiome(s) | | Maternal OR mother OR woman OR women OR female | Stress OR anxiety OR “mental health” OR “emotional health” OR “psychosocial stress” OR “mood disorders” | Microbiome OR Microbiota OR “Gut Microbiome” OR “Gut microbiota” OR “intestinal microbiota” OR “gastrointestinal microbiome” OR "gastrointestinal microbiota" OR flora OR metagenomics OR biofilms OR dysbiosis  and microflora | Prenatal OR antenatal OR antepartum OR Pre-birth OR Perinatal OR Prepartum OR Peri-partum OR Pregnancy OR preg* OR Gestation OR Postnatal OR Postpartum |
| 1. Maternal stress and human milk microbiome | | Maternal OR mother OR woman OR women OR female | Stress OR anxiety OR “mental health” OR “emotional health” OR “psychosocial stress” OR “mood disorders | "human milk microbiome" or "milk microbiome" or "HMM" or “breastmilk microbiome” OR "human milk composition" | Prenatal OR antenatal OR antepartum OR Pre-birth OR Perinatal OR Prepartum OR Peri-partum OR Pregnancy OR preg* OR Gestation OR Postnatal OR Postpartum |
| 1. Interplay of all concepts   Maternal stress, maternal and/or infant gut microbiome, and human milk microbiome | | Maternal OR mother OR woman OR women OR female | Stress OR anxiety OR “mental health” OR “emotional health” OR “psychosocial stress” OR “mood disorders | Microbiome OR Microbiota OR “Gut Microbiome” OR “Gut microbiota” OR “intestinal microbiota” OR “gastrointestinal microbiome” OR "gastrointestinal microbiota" OR flora OR metagenomics OR biofilms OR dysbiosis  OR microflora | Prenatal OR antenatal OR antepartum OR Pre-birth OR Perinatal OR Prepartum OR Peri-partum OR Pregnancy OR preg* OR Gestation OR Postnatal OR Postpartum |
|  |  |  |  | "human milk microbiome" or "milk microbiome" or "HMM" or “breastmilk microbiome” OR "human milk composition |  |
