# Supplementary material for: The impact of perinatal maternal stress on the maternal and infant gut and human milk microbiomes: A scoping review
Source: PLoS One. 2025 Feb 28;20(2):e0318237. doi: 10.1371/journal.pone.0318237 (PMC11870360; doi:10.1371/journal.pone.0318237)
Supplement: Attachment — Submitted filename: Response to reviewers comments Draft 1 a.docx [file pone.0318237.s005.docx]

**The impact of perinatal maternal stress on the maternal and infant gut and human milk microbiomes: A scoping review**

The authors would like to thank the reviewers of PLOS one for their valuable feedback regarding the manuscript. As requested, below are our point-by-point responses to the recommendations made by reviewer 1 & 2 and the page within the revised manuscript where the corresponding changes appear highlighted in track changes.

| **Comment** | **Reply** |
| --- | --- |
| **Reviewer 1** |  |
| *“*The statistical analyses seem to be handled appropriately, and the rigor is evident. That said, I did notice some significant heterogeneity among the included studies, which could impact the generalizability of your findings. It might be helpful to delve a bit deeper into how this heterogeneity could influence the overall conclusions” | Heterogeneity and inconsistencies among both stress measurement and the microbiome collection are detailed in pg. 24/25 and 25/26 however I agree it could be more clearly highlighted in relation to influencing overall conclusions.  Amended in discussion page 25.  There is clear heterogeneity across these studies, particularly in terms of the data collection of maternal stress. These include the types of subjective and/or objective measurement tools, exposure of acute vs chronic stressors within various timelines, along with inclusion of different confounding factors. As a result, caution is needed when interpreting the findings and developing overall conclusions as these inconsistencies may limit their generalisability. For example, over or under estimation of stress is possible and its link to symbiosis or dysbiosis of the microbiome be it due to single data collection points potentially missing an acute or chronic stress, or the presentation of the status of the microbiome at various timelines. These inconsistencies make it difficult to reach an overall conclusion on how stress impacts the microbiome across the studies in the review.  However, heterogeneity can also be seen as beneficial in this review, as it highlighted the value of incorporating objective data when assessing the physiological effects of stress on the microbiome. In this context, as with much psychological research, it may reflect the depth of understanding of the subject under investigation (Linden & Hönekopp, 2021). |
| I think the discussion could benefit from a bit more depth, particularly when it comes to the methodological challenges and inconsistencies across the studies. Addressing these issues more thoroughly could provide readers with a better understanding of the current state of research in this area and offer clearer guidance for future studies | Amended to add a paragraph to the discussion: Pg 27  This research, which involves using various tools to measure the complex concept of stress and multiple biological collection methods for both mother and infant over different timelines, highlights significant methodological challenges for research teams. Although only seven studies were included in the review, the variability in methods highlights the diverse approaches that can be taken, especially when dealing with the variable of stress. Only three of the studies combined subjective and objective data, with less than half using multiple data collection points.  Many studies were excluded from this review due to the inability extrapolate data from anxiety and depression scales, highlighting the difficulty of untangling the multifaceted nature of stress(Cohen et al., 1997). Despite ongoing debates in the literature, there is no definitive guide dance on the reliable number of time points for stress data collection to differentiate between acute and chronic stress, nor is there a universally accepted questionnaire or biomarker assessment (Cohen et al., 1997; Dorsey et al., 2022; Nast et al., 2013; Noushad et al., 2021). Ultimately, these decisions often depend on the researchers' discretion of how they define stress. Similarly, in microbiome research, there is ongoing debate about whether cross-sectional samples are sufficient to represent the microbiome's composition, even when 16S sampling methods are consistently applied throughout the studies in the review (Lyu et al., 2023).  Limitations and inconsistencies can arise from variability in data collection time points due to limited access for sampling mothers and infants during the perinatal period. Maintaining participant engagement, especially pre- and postnatally, is also challenging, often resulting in smaller sample sizes that weaken findings—five of the studies included had fewer than 85 participants. Additionally, collecting multiple biological samples from both mother and infant across various locations (clinics, hospitals, and homes) presents logistical challenges, including timing and storage of samples (Lyu et al., 2023; Noushad et al., 2021). There is considerable variability in confounding factors across all cohorts, including mothers, infants, and the microbiome, leading to inconsistencies between studies. Only some authors acknowledge not accounting for all confounders. The varied tools used results in different analysis of data sets again complicating synthesising overall findings. Acknowledging and addressing some of these methodological challenges can assist progression of research in this novel topic area. |
| **Reviewer 2:** |  |
| Introduction:  Consider addressing the impact of maternal nutrition on maternal and infant microbiomes during the perinatal period. Maternal diet is a critical factor that can influence the composition and function of the microbiome, potentially affecting maternal health, pregnancy outcomes, and infant development. | This review focuses on perinatal maternal stress as the main concept however, it acknowledges in the introduction there is many maternal factors such as diet, body mass index, mode of delivery, etc that are significant to the human milk microbiome that are beyond the scope of this review.  *“However, recent evidence identifies the human milk microbiome is susceptible to maternal factors of body mass index, diet, mode of delivery, general health, and lactation stage, that can also influence the microbial seeding of an infant* (Di Benedetto et al., 2020; Gay et al., 2018; Kortesniemi et al., 2021; Mantziari & Rautava, 2021)*.”*  Amendments as suggested Pg 4 .  *Research has advanced the understanding of the gut microbiome's role in health and disease, including* ***many various*** *factors influencing maternal and infant gut microbiomes and the human milk microbiome.*  Additional amendment as requested:  A recent systematic review, for example, have investigated the crucial role of maternal diet quality in influencing the gut microbiome profile and diversity of both the mother and infant that can also influence infant development (Maher et al., 2023). |
| Methods :   a) Please include a discussion on the quality assessment of the included studies b) Consider addressing potential biases such as selection bias, publication bias, and reporting bias, and their possible influence on the review’s results. | a. The objectives of the scoping review were to identify: 1. the methods and tools used by which stress and the microbiome(s) are measured 2. the impact of perinatal stress on specific taxa in the microbiome(s): 3. the impact of perinatal stress on microbiome(s) diversity 4. any gaps in the evidence.   This scoping review is not designed to inform clinical decision making but to provide an overview of evidence regarding the above objectives. Assessment of methodological quality or risk of bias is often not a mandatory step for a scoping review, it is not included within JBI guidelines and was not included in this review. (Munn et al., 2018; Peters et al., 2020, 2021; Peterson et al., 2017).   b. Amendment to Data Extraction in Methods Pg 9 :   There is no structured risk of bias assessment as it was not required to meet the objectives of this scoping review. However, Daudt et al.(2013) recommend assessing research quality before charting. Therefore, the study selection and data extraction process considered potential bias in design, conduct, and analysis. The data extraction table clearly identifies sample, setting, measurement of data, confounding factors and statistical analysis completed**.**  Amendment to Discussion ( pg.27 )   c. In view of such as a small number of studies post screening, publication bias needs to be considered particularly where the possibility of statistically insignificant studies did not get published |
| Results and discussion :   a) Explain how the findings might be applied in clinical settings. Consider how the findings of your review might influence guidelines for prenatal care, particularly regarding stress management b) Indicate whether a sensitivity analysis was considered or conducted to evaluate the robustness of the findings. c) Consider addressing the variability in how studies define types of stress (e.g., psychological vs. physical, acute vs. chronic) and the tools used to measure stress (e.g., self-reports, physiological measures). d) Discuss the potential confounding effects of comorbid conditions (e.g., depression, anxiety, or physical health issues) that could influence both maternal stress and microbiome composition. | Amendments as follows:   a) Insert to end of Discussion pg 29:   This review identifies that perinatal maternal stress significantly impacts the abundance and diversity of key bacteria in both maternal and infant gut microbiomes. This emphasises the importance of addressing perinatal stress for the well-being of both mother and infant. Since stress is a modifiable factor, these findings highlight the importance of prioritizing clinical interventions to prevent and manage stress as a key aspect of health promotion, both prenatally and postnatally in healthcare. By tackling perinatal stress, immediate risks such as low birth weight and preterm delivery could be reduced, while also fostering stronger mother-child bonding during post-natal periods (Littleton et al., 2010; McCarthy et al., 2021; Van den Bergh et al., 2005). Furthermore, addressing maternal stress may mitigate negative behaviours like smoking and alcohol consumption thus further improving lifestyle (Matvienko-Sikar et al., 2021; Westerneng et al., 2017). Moreover, a “homeostatic” or stable infant gut microbiome has the potential to reduce risk to chronic metabolic, immunological and neurological disease(Grech et al., 2021; Gritz & Bhandari, 2015; Rotem-Kohavi et al., 2021; Turroni et al., 2020).   b) A sensitivity analysis was not conducted or considered mainly due to the fact that this is a scoping review with no decisions or missing information within the studies which could influence robust findings, which is the case of more focused systematic reviews. c) The choice of tools to measure stress are detailed in the data extraction table. The variability of the use of subjective self-report versus the use physiological biomarkers or both and how they influence the results in the microbiome are discussed on page 23/24. Addressing this variability is also discussed in future research on pg.28.   However, a clearer identification of the inconsistencies and heterogeneity and the influence of this on results will be amended in discussion and added on pg. 27  There is clear heterogeneity across these studies, particularly in terms of the data collection of maternal stress. These include the types of subjective and/or objective measurement tools, exposure of acute vs chronic stressors within various timelines, along with inclusion of different confounding factors. As a result, caution is needed when interpreting the findings and developing overall conclusions as these inconsistencies may limit their generalisability. For example, stress may be over- or underestimated, and its link to microbiome symbiosis or dysbiosis could be missed due to single data collection points, which might overlook acute or chronic stress, or because the microbiome's status is measured at varying timelines. These inconsistencies make it difficult to reach an overall conclusion on how stress impacts the microbiome overall across the studies in the review.  However, heterogeneity can also be seen as beneficial in this review, as it highlighted the value of incorporating objective data when assessing the physiological effects of stress on the microbiome. In this context, as with much psychological research, it may reflect the depth of understanding of the subject under investigation (Linden & Hönekopp, 2021).   d) This review excluded anxiety and depression unless data on stress could be extrapolated and analysed. The rationale for this was that anxiety and depression can often be an enduring mental health illness with a clinical diagnosis and medication required(American Psychiatric Association, 2013), thereby adding more complex variables to the Population, concept and context. The focus for this review was stress of a healthy mother, or “psychosocial stress” therefore the discussion of such co-morbidities was beyond the scope of this review. |
|  |  |

**Journal Requirements:**
